# Transfusion management of severe anaemia in African children: a consensus algorithm

**DOI:** 10.1111/bjh.17429

**Published:** 2021-05-06

**Authors:** Kathryn Maitland, Sarah Kiguli, Peter Olupot-Olupot, Robert O. Opoka, Yami Chimalizeni, Florence Alaroker, Sophie Uyoga, Dorothy Kyeyune-Byabazaire, Bridon M’baya, Imelda Bates, Thomas N. Williams, Deogratias Munube, Dora Mbanya, Elizabeth M. Molyneux, Annabelle South, A. Sarah Walker, Diana M. Gibb, Elizabeth C. George

**Affiliations:** 1 Department of Infectious Disease, Division of Medicine, Institute of Global Health and Innovation, Imperial College, London, UK; 2 Department of Paediatrics and Child Health, School of Medicine, Makerere University and Mulago Hospital, Kampala; 3 Faculty of Health Sciences, Busitema University, Mbale Regional Referral Hospital, Mbale, Uganda; 4 College of Medicine, Malawi-Liverpool-Wellcome Research Programme, Blantyre, Malawi; 5 Soroti Regional Referral Hospital, Soroti, Uganda; 6 Kenya Medical Research Institute (KEMRI)/Wellcome Trust Research Programme, Kilifi, Kenya; 7 Uganda Blood Transfusion Services (BTS), National BTS, Kampala, Uganda; 8 Malawi BTS, Blantyre, Malawi; 9 Liverpool School of Tropical Medicine, Liverpool, UK; 10 Haematology & Transfusion Service, Centre Hospitalier et Universitaire, Yaounde, Cameroon; 11 Medical Research Council Clinical Trials Unit (MRC CTU), University College London, London, UK

**Keywords:** anaemia, African children, transfusion, guidelines, malaria

## Abstract

The phase III Transfusion and Treatment of severe anaemia in African Children Trial (TRACT) found that conservative management of uncomplicated severe anaemia [haemoglobin (Hb) 40–60 g/l] was safe, and that transfusion volume (20 vs. 30 ml/kg whole blood equivalent) for children with severe anaemia (Hb <60 g/l) had strong but opposing effects on mortality, depending on fever status (>37·5°C). In 2020 a stakeholder meeting of paediatric and blood transfusion groups from Africa reviewed the results and additional analyses. Among all 3196 children receiving an initial transfusion there was no evidence that nutritional status, presence of shock, malaria parasite burden or sickle cell disease status influenced outcomes or modified the interaction with fever status on volume required. Fever status at the time of ordering blood was a reliable determinant of volume required for optimal outcome. Elevated heart and respiratory rates normalised irrespective of transfusion volume and without diuretics. By consensus, a transfusion management algorithm was developed, incorporating three additional measurements of Hb post-admission, alongside clinical monitoring. The proposed algorithm should help clinicians safely implement findings from TRACT. Further research should assess its implementation in routine clinical practice.

## Introduction

In sub-Saharan Africa, children admitted to hospital with severe anaemia [defined as a haemoglobin (Hb) of <60 g/l] present a major public health burden on in-patient and blood transfusion services. Although timely transfusion is frequently lifesaving, equitable access to adequate supplies of safe blood remains a key challenge.^1^ In 2013 the World Health Organization (WHO) Global Database on Blood Safety reported that <5 units of blood were donated per 1000 people in most African countries,^2^ far fewer than estimated requirements of 20 units/1000 per year.^2^ Despite considerable investment by external funders in the decade since their previous report (2004–2005),^3^ few countries had made significant improvements in their collection rates.

Guidelines developed by the WHO encourage the conservative use of blood in children, restricting routine transfusion to those with severe anaemia, defined as an Hb <40 g/l, or 40–60 g/l with clinical signs of severity.^4^ However, the underpinning evidence base for this recommendation is weak^5^ and adherence to these guidelines is poor,^6,7^ which is compounded by inconsistent Hb threshold recommendations for transfusion for malaria. Currently, 20 ml/kg of whole blood (or 10 ml/kg of packed cells) is recommended for severe anaemia.^4^ However, if standard formulae for transfusion volume were applied,^8,9^ the one-size-fits-all 20 ml/kg recommendation would underestimate transfusion requirements by ~30%.^6,10^ A consensus international guideline on transfusion of critically ill children also noted a weak global evidence base and specifically highlighted the need to evaluate optimal transfusion volumes.^11^

The Transfusion and Treatment of Severe Anaemia in African Children Trial (TRACT) was designed as a multicentre trial to establish transfusion and treatment strategies in sub-Saharan Africa and investigated: (i) whether an immediate transfusion in children with uncomplicated severe anaemia (Hb 40–60 g/l) would improve outcomes and (ii) whether 30 *versus* 20 ml/kg whole blood might improve outcomes, defined as mortality to day 28 (primary outcome) and day 180, and the need for additional transfusions and relapse of severe anaemia.^12^

The trial results, published in two linked manuscripts in 2019,^13,14^ showed that an immediate transfusion is not required in children with uncomplicated severe anaemia (Hb 40–60 g/l without severity features), as long as children are monitored for progression to the development of severe and/ or complicated anaemia during their initial hospital stay; such progression occurred in 49% of trial participants who then received a transfusion.^13^ The results also showed that giving larger volumes of blood, 30 ml/kg whole blood equivalent ver*sus* the 20 ml/kg recommended in the current WHO guidelines, to children with severe anaemia who did not have a fever (axillary temperature ≤37·5°C, measured at screening) halved the number of deaths by 28 days [hazard ratio (HR) 0·43, 95% confidence interval (CI) 0·27–0·69].^14^ However, the opposite occurred in children who were febrile (>37·5°C) at screening; 30 ml/kg whole blood equivalent almost doubled the risk of death in comparison to children who received the lower, 20 ml/kg, volume (HR 1·91, 95% CI 1·04–3·49; *P* value for heterogeneity of effect on mortality *P* = 0·00009).

The TRACT results provide an evidence base with potential to improve outcomes for children with severe anaemia. Furthermore, initial economic analyses also suggested that if the results were implemented and adhered to by clinicians, there could be substantial cost savings for blood transfusion services. To explore this further, the TRACT investigators shared results from the trial with key stakeholders at a meeting in Uganda in February 2020, co-hosted by the African Society for Blood Transfusion and the Ugandan Paediatric Association. The meeting was attended by stakeholders from 12 sub-Saharan countries and included representatives from blood transfusion services, health service providers (including paediatricians involved in providing continued professional development and training courses) and representatives from international policymakers, including the WHO and Médecins Sans Frontières (Appendix 1). Our aim was to clarify current practice in different settings within Africa, to discuss the implications of the TRACT findings from a stakeholder perspective, to identify potential barriers to their uptake and to identify any additional requirements that might be needed to support their safe implementation, including further analyses of the TRACT data.

A major output from discussions at this stakeholder meeting was the development of a transfusion management algorithm for future implementation, which was agreed by consensus. Here, we present the algorithm and the additional analyses that support it.

## Methods

A summary of the trial and statistical methods is available in the supplemental appendix. Blood transfusions were supplied by the local blood transfusion services (BTS), which included three blood component types: (i) whole blood (WB), collected from donors and stored without any preparation or via transfer bags into smaller bags of whole blood (known as ‘Pedi-Packs’); (ii) packed red cell concentrates (RCC), produced by centrifugation to remove platelets and plasma, followed by the addition of sodium, adenine, glucose and mannitol solution; and (iii) RCC created using gravity (Uganda only).^15^ To support the algorithm, we investigated detailed response to transfusion volume and requirement for additional transfusions. At the request of participants at the meeting we provided original and further analyses of the primary outcome (28-day mortality) in the following key subgroups: (i) nutritional status (protocol defined); (ii) malaria status including those with high malaria burdens (plasma *Plasmodium falciparum* histidine-rich protein 2 (PfHRP2) concentration of >1000 ng/ml^16^); (iii) children with shock defined by the Fluid Expansion As Supportive Therapy in critically ill African children (FEAST) trial eligibility criteria;^17^ and those with sickle cell disease (SCD). These subgroups all have direct implications for the current WHO management guidelines.^4^ Finally, additional exploration of the fever/no fever interaction was undertaken for a number key subgroups where the interaction might potentially have been impacted for physiological reasons [including SCD and Blackwater fever (haemoglobinuria)].^18^

## Results

**Fig 1:**
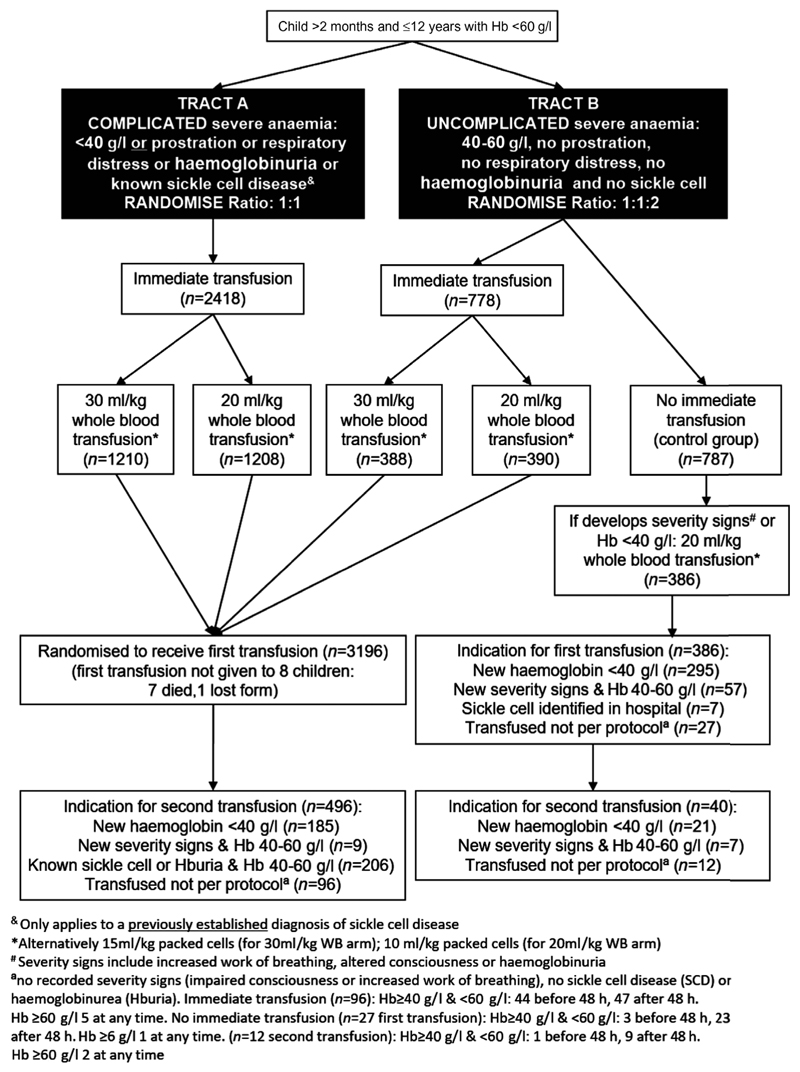
Transfusions given in the TRACT trial.

**Fig 2:**
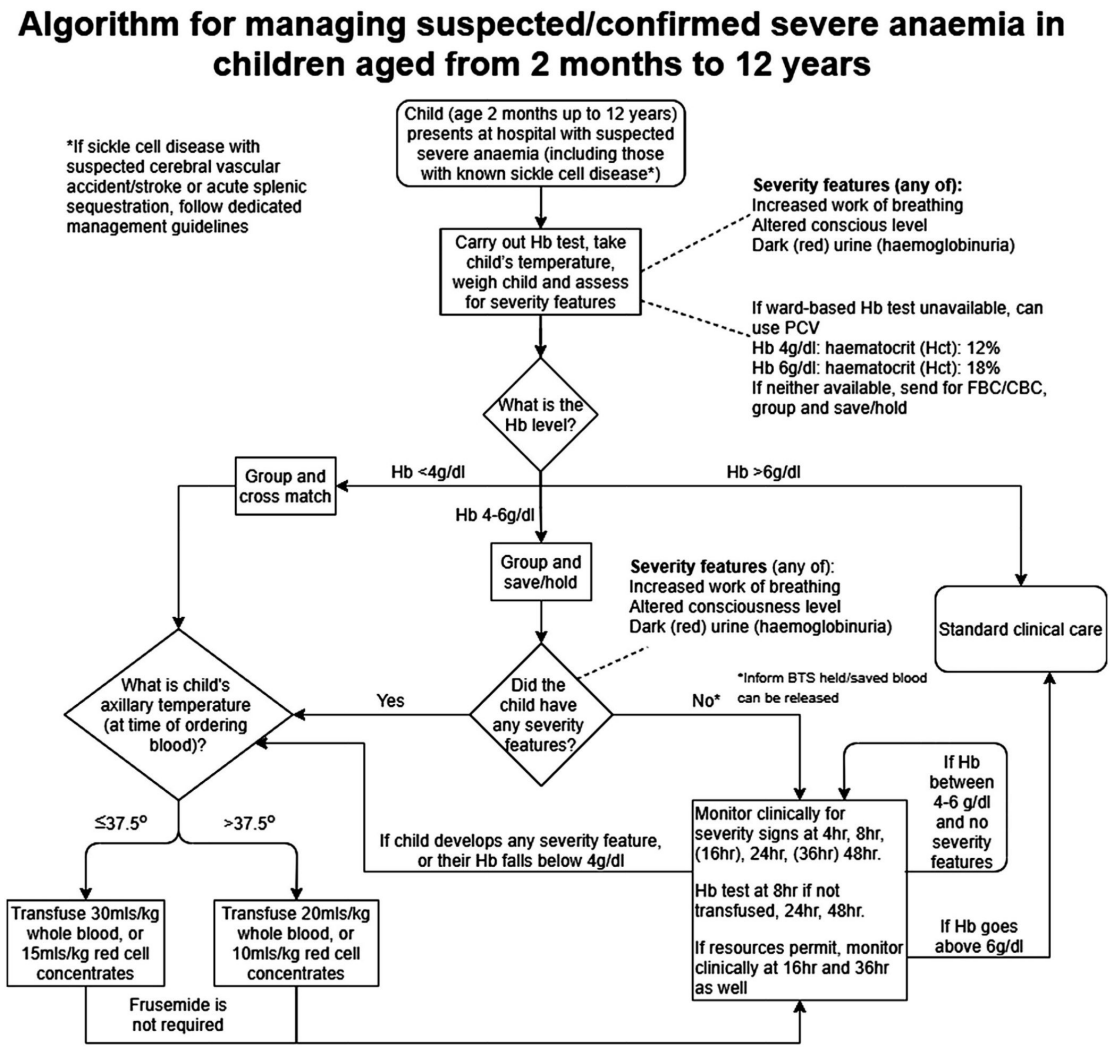
Algorithm for managing suspected/confirmed severe anaemia.

**Fig 3:**
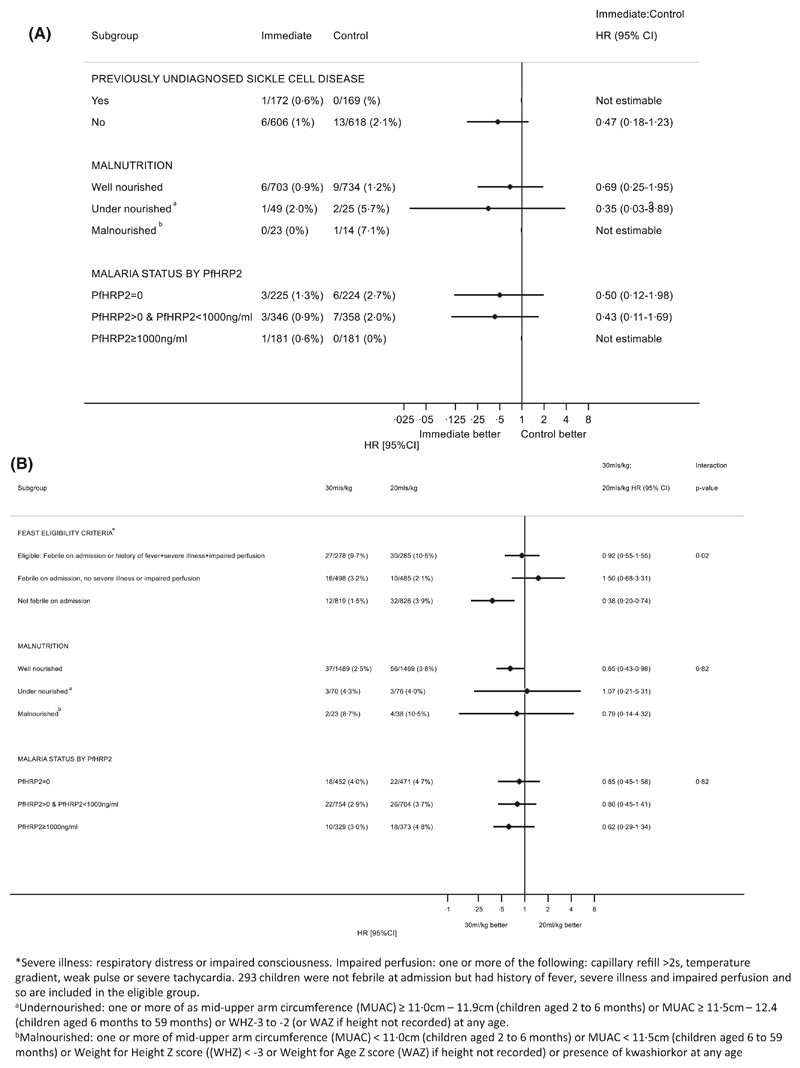
(A) Morality at 28 days by subgroups for the immediate vs control timing of transfusion comparison. (B) Morality at 28 days by subgroups for the transfusion volume comparison. PfHRP2, plasma *Plasmodium falciparum* histidine-rich- protein 2.

The TRACT recruited children aged 2 months to 12 years presenting to hospital with both uncomplicated and complicated severe anaemia (Fig 1), and the algorithm is aimed at this population (Fig 2). We found no evidence to show that outcome was affected by nutritional status (including children classified as undernourished) or by SCD status (which was unknown at the time of randomisation in the majority) (Fig 3A). In the current WHO guidelines, malaria hyperparasitaemia is an indicator for immediate transfusion.^4^ We therefore assessed whether this was supported by the TRACT. Instead of ‘hyperparasitaemia’, we used the value PfHRP2 ≥1000 ng/ml, a parameter that more accurately reflects the highest total parasite burden, and which distinguishes ‘true’ severe malarial anaemia from anaemia in the presence of incidental parasitaemia.^16^ We found that outcome was the same in children managed on a deferred transfusion protocol as in those receiving immediate transfusion (Fig 3A). Thus, in our algorithm we did not provide separate recommendations with regard to immediate or deferred transfusion for these groups.

For the higher *versus* lower volume comparison, the stake-holder meeting group identified two subgroups that could, potentially, have increased the risk of a worse outcome with higher transfusion volumes, irrespective of fever status: (i) children with clinical features of shock (severely impaired perfusion as defined in FEAST trial^17^); and (ii) children with anthropometric features of undernutrition. As shown in Fig 3B none of these subgroups had outcomes that differed significantly from the overall findings. Therefore, the stakeholder meeting concluded that the recommendations on transfusion volume did not need to differ according to a child’s perfusion status, nutrition status or malaria parasite burden.

The initial evaluation of a child with suspected severe anaemia should include a Hb test, axillary (or other) temperature, weight and assessment for severity signs (altered conscious level or respiratory distress). Then the child can be grouped into one of three categories: (i) severe and complicated anaemia (Hb <40 g/l or Hb 40–60 g/l, with clinical severity features); (ii) Hb 40–60 g/l, without clinical severity features; and (iii) Hb >60 g/l, with or without severity signs. The first group require immediate transfusion; the second group do not require immediate transfusion but should have a blood sample sent to the transfusion services for blood ABO grouping^19^ and blood unit(s) (WB or RCC) saved while being monitored both clinically and for Hb level (at a minimum of 8, 24 and 48 h following identification). The volume for transfusion, based on weight and temperature can be obtained from Table I. The third group does not require transfusion (with Hb >60 g/l) and can be managed without further Hb monitoring (Fig 2). Once children have been transfused, or for stable children with Hb 40–60 g/l, they will continue to be monitored and further transfusion management will depend on their latest Hb and/or clinical features.

### Blood volume dosing table

In the TRACT, we were reassured that >96% of children received the correct volume of blood. Children weighed between 5 and 35 kg, the majority (~75%) weighing ≤15 kg. Thus, most of the WB prescriptions for the 20 ml/kg strategy were between 100 and 320 ml and for the 30 ml/kg strategy were between 150 and 480 ml. To simplify future implementation of the algorithm, we have generated a simplified ‘lookup’ chart together with bag size recommendations for WB Pedi-Packs for appropriate weight bands (Table I Blood dosing chart). If WB is unavailable then the equivalent RCC doses are half the doses stated in the ‘look-up’ chart. This is intended to be used alongside the transfusion algorithm.

### Timing and stability of fever

For children with severe or complicated anaemia, the volume of blood component ordered (20 or 30 ml/kg WB) in the algorithm is dependent on the axillary temperature at the time of requesting blood. For those with uncomplicated anaemia that subsequently need a transfusion, the axillary temperature at the time of ordering blood would be used. In the TRACT trial nearly all children (3106/3196, 97·2%) had a history of fever in the current illness, but only 1253 (39·2%) had a recorded fever at screening, before the receipt of a transfusion. Screening temperature is only at one time point, so we examined the patterns of fever during their hospital stay and found only 10% of children without a fever at screening developed one after the transfusion had started. For children with a fever at screening, the fever had waned in 80% by 16 h (Fig 5). The heterogeneity in the estimate of mortality remained for groups defined by their fever status over 8 h (Fig 5; Figure S2) with benefit from 30 ml/kg for those with no fever at screening, even if they had intermittent fever during the subsequent 8 h. However, if a child subsequently required new or additional transfusion then the group recommended that the axillary temperature at the time of requesting blood would be used to indicate volume required, rather than the initial fever status at initial screening.

### What might be the mechanisms for excess mortality in children receiving 30 ml/kg with fever?

We found no strong evidence implicating any specific cause for the excess mortality associated with fever and blood volume. Neither known SCD nor haemoglobinuria at admission had any effect on the fever/transfusion volume interaction, nor did the age of the child (Figure S1). Transfusion-related volume overload [pulmonary oedema, transfusion-related lung injury (TRALI) or transfusion-associated cardiac overload (TACO)] was ruled out because of the small number of solicited events (n = 5) in the trial (summarised in Table SI); only one was adjudicated to be probably related to blood volume. No child received diuretics or other anti-failure medications. The recovery of elevated heart and respiratory rates occurred similarly in both febrile and afebrile groups (Figure S3). Blood component type (WB or RCC) was also explored in a pre-planned sub-analysis of the TRACT and was found not to have contributed to adverse outcomes. The stakeholders supported the algorithm recommending both 30 and 20 ml/kg transfusion WB volumes (or RCC equivalent), depending on fever status.

### Children in the no immediate transfusion category: what triggered transfusion and when was it given?

**Fig 4:**
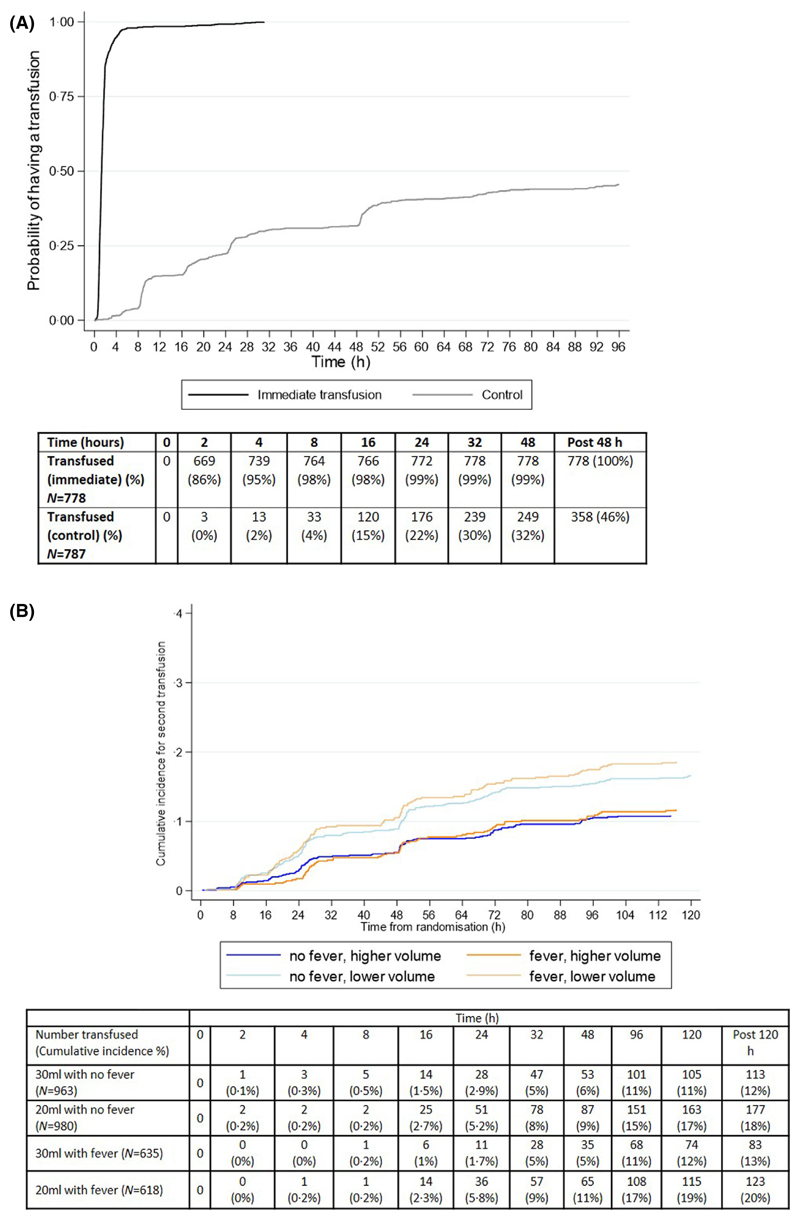
(A) Time to Transfusion in Immediate vs Control Comparison. (B) Cumulative incidence in second transfusion in the 30 mls/kg vs. 20 mls/kg split by fever and no fever. Note: A competing risks analysis with discharge, absconding and death as competing events. There are 39 transfusions given after 120 h from baseline not included on the graph. [Colour figure can be viewed at wileyonlinelibrary.com]

Children with uncomplicated severe anaemia (no severity signs and Hb 40–60 g/l) would be monitored clinically according to the algorithm, with a Hb test at 8, 24 and 48 h and not transfused unless they develop severity signs or Hb <40 g/l. In the TRACT trial 386/787 (49%) children randomised to not receive an immediate transfusion, subsequently did receive one; details of timing of triggered transfusions are in Figs 1 and 4A. Haemodilution, by liberal use of intravenous fluids, was discounted, based on the fact that no fluid boluses were given^17^ and children only received maintenance fluids until they were able to drink. Compared to children randomised to immediate transfusion, who received blood at a median [interquartile range (IQR)] of 1·3 [0·9–1·7] h after randomisation, 295/386 (76%) of those in the deferred group received a subsequent transfusion, principally triggered by a fall in Hb to <40 g/l, at a median (IQR) of 24·9 (9·2–49·8) h after randomisation. Reasons for transfusion in an additional 57 (15%) children were the development of clinical severity features (impaired consciousness or increased work of breathing) and SCD identified in hospital (n = 7; 2%). Only 27 (7%) had a transfusion without a very low Hb or a recorded severity feature, largely after 48 h when reasons for transfusion were not routinely captured. Of note, the timing of triggered transfusions largely coincided with the routine Hb assessment in the trial (at 8, 16, 24 and 48 h after randomisation) and 49% were given within 24 h (excluding the 24 assessment) (Fig 4A). A further two Hb measurements (at 36 and 48 h) would have identified around two-thirds (249/386; 64·5%) of children in the control group who subsequently received a transfusion. Thus, this informed the recommendation in the algorithm for repeat Hb measurements at 8, 24 and 48 h (Fig 2), with additional measurements at 16 and 36 h where resources permit.

### Timing of deaths in controls: concern that this may be related to delaying transfusion

Of the 13 children in the control arm who died, 10 had received a transfusion prior to death and these were received at a median of 9 h after randomisation.^13^ Furthermore, of seven children transfused before 10 h, one died on Day 1 (who had a screening Hb of 59 g/l) and six died between days 4–23, with a recorded 48-h Hb level of 50–90 g/l (only two were <60 g/l). Three were not transfused; one died on day 1 with last recorded Hb of 46 g/l, while two died on day 6 and day 25 with 48-h Hb levels of 54 and 65 g/l respectively. In summary, delay in transfusion or no receipt of transfusion does not appear to have made a clear contribution to outcome among these deaths (Supplemental material original report).^13^

### In children requiring additional transfusions: what triggered re-transfusion events and when were they given?

In the TRACT trial, of those receiving immediate transfusion, 3188/3196 (99%) received one, and 496/3196 (15·5%) received a second transfusion, 300 (19%) in the 20 ml/kg group and 196 (12%) in the 30 ml/kg group, an absolute difference of 6% (95% CI 4–9%, *P* < 0.0001) greater in the 20 ml/kg strategy.^14^ Overall, re-transfusions were due to falls in Hb to <40 g/l in 185 (37%) children, and occurred more frequently in the 20 ml/kg group (42% vs. 30% in 30 ml/kg group) (Table II), or it was felt that clinically stable children with SCD or haemoglobinuria with Hb between 40 and 60 g/l needed it corrected to >60 g/l (n = 206, 42%). The number for which an Hb <40 g/l prompted the second transfusion supports our recommendation in the algorithm for repeat Hb measurements at 8, 24 and 48 h among children who have been transfused, as well as those being monitored before a possible transfusion. These three additional measurements capture the large proportion of repeat transfusions, as very few received an additional transfusion for new clinical signs of severity after admission (2%) or when Hb was >60 g/l (Fig 1). With regard to the timing of additional transfusions, they occurred earlier and at a higher rate in those randomised to 20 ml/kg. Approximately 5% of children in this group received a transfusion by 24 h and 9–10% by 48 h, compared to under half this rate at both time points in the 30 ml/kg arm (Fig 4B). Although it was the second most common reason for a second transfusion, SCD status (known or unknown) was not a risk factor for poor outcome, this group was not segregated in the algorithm for repeat transfusion.

### Consensus blood transfusion algorithm

Following discussion of all of the above, the meeting participants endorsed the proposed paediatric blood transfusion algorithm based on the evidence provided by the TRACT (Fig 2). The key elements start with triage of a child with suspected severe anaemia, incorporating an assessment of severity (to determine the need for a transfusion), with further management guided initially by an assessment of Hb (or haematocrit if Hb is not possible) and fever status at the time a blood transfusion is ordered from the blood transfusion service, in order to determine whether to request a higher volume of blood (30 ml/kg WB or 15 ml/kg RCC), or standard volume (20 ml/kg WB or 10 ml/kg RCC) if fever is present. Informed by the main time points when first (for children with uncomplicated anaemia) or repeat transfusions occurred, our proposed algorithm also incorporates a minimal number of additional measurements of Hb (or haematocrit) and clinical monitoring.

## Discussion

If fully implemented, our proposed paediatric blood transfusion algorithm could avert the need for immediate transfusion in children with severe uncomplicated anaemia (including those with SCD) and provide higher volumes of blood to children with severe and complicated anaemia (based on clinical signs and severity of anaemia) who do not have a fever. Both these strategies would improve clinical outcomes, more specifically, in children without a fever, a liberal 30 ml/kg strategy would result in almost 60% fewer children dying, while for children with a fever a conservative 20 ml/kg strategy would prevent a 50% higher mortality were they to receive the higher volume. Although more complex, the TRACT showed that this approach could substantially reduce the demand for paediatric transfusion because the higher volumes given to children without fever would also significantly reduce the number of additional transfusions required. Our previous cost-effectiveness analysis indicated substantial savings for health services, with 60% reduction in blood transfusion required compared to immediate transfusion for all children with uncomplicated severe anaemia (Hb <60 g/l).^13^ The unadjusted cost-effectiveness analyses, presented in the trial reports, demonstrated that the ‘no immediate transfusion strategy’ would cost $66·46 (American dollars) per child in comparison to $72·09 per child for an immediate transfusion strategy: the Meeting attendants recognised that the cost savings would be even greater where per-unit costs of donor blood are higher.

It was recognised that implementation of the algorithm in a real-life setting is required, in order to establish feasibility, impact on mortality and whether it reduces unwarranted requests for paediatric transfusions. However, most paediatricians felt that the proposed recommendations for Hb monitoring could be a substantial hurdle for clinical services in some resource-limited hospitals (see accompanying Perspective Paper). As demonstrated in the cost-effectiveness models, major costs to health services are the ‘hotel costs’ (days in hospital) and the price of a blood transfusions. Total costs could be reduced by access to 24-h point-of-care Hb measurements, which would require advocacy to the health services and policy makers from both paediatricians and blood transfusion services.

The strengths of the TRACT trial include its size, multicentre nature (Uganda and Malawi), broad eligibility criteria that enhances its generalisability and enables incorporation of large subgroups with malaria and SCD. In children with uncomplicated severe anaemia, we found a large group of children with undiagnosed SCD. We found no evidence that not transfusing these children (as they did not develop severity features or profound anaemia) resulted in a worse outcome (mortality to Day 28 and Day 180). On this basis, children with either diagnosed or undiagnosed SCD without severity features can be safely managed using a triggered transfusion strategy and thus incorporated into the algorithm.

**Fig 5:**
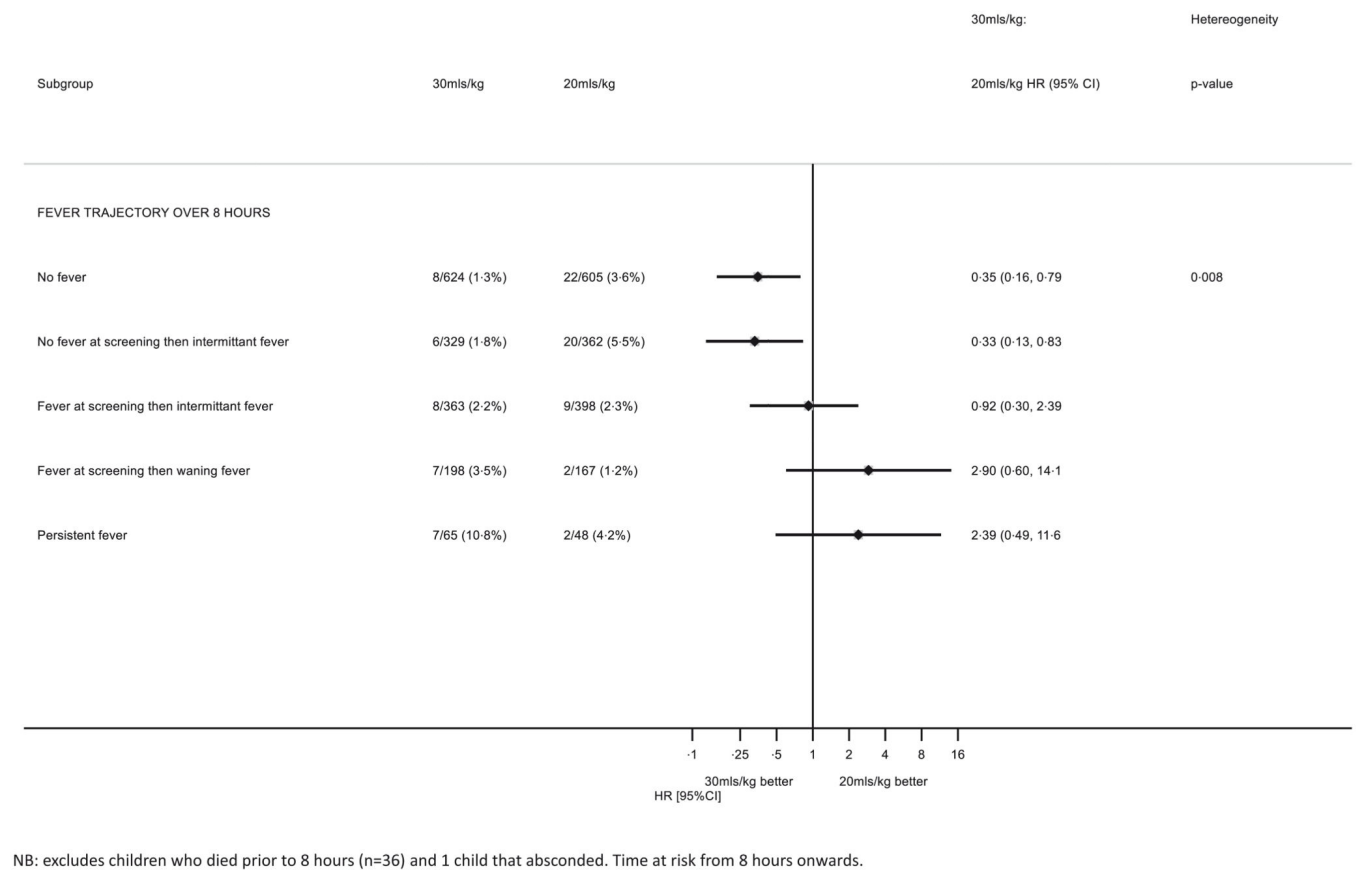
Justification for transfusion volume management based on initial temperature and not on subsequent temperatures.

As noted by the meeting stakeholders most transfusions in the TRACT were given very shortly after screening/identification of a case of severe anaemia (when axillary temperature was recorded), which may not be replicable in usual clinical practice. The relevance of a temperature taken hours before a blood transfusion becomes available was explored by additional analyses. These supported the recommendation that at the time that blood is ordered for a child who has no fever at that time, even if they subsequently developed an intermittent fever (Fig 5), it was still safe (and beneficial) to transfuse with a higher volume. Similarly, for children who have a fever at the time blood is ordered and which then wanes, there is no evidence to support that the requested 20 ml/kg volume would lead to any difference in outcome compared to 30 ml/kg. For children with a persistent fever prescribing 20 ml/kg is also supported by these analyses.

## Conclusions

The new evidence provided by the TRACT could lead to important refinements of the WHO transfusion guidelines. First, standardising the definition of severe anaemia, we suggest an Hb <60 g/l. Second, clarifying the definitions of uncomplicated severe anaemia, and severe and complicated anaemia, by using clinical signs of severity. Third, a strong recommendation (based on high quality of evidence) that children with uncomplicated severe anaemia do not require immediate transfusion, irrespective of the underlying causes, but do require monitoring because ~50% will develop severe and life-threatening anaemia requiring subsequent transfusion. Fourth, new recommendations to include a minimal number of clinical and Hb monitoring reviews in a child with severe anaemia (both uncomplicated and complicated) in order to identify those needing an initial or additional transfusion. Fifth, new recommendations on the volume of blood to transfuse, according to fever status when a blood transfusion is ordered, and that either WB or packed RCC (at half the volume) can be used. Finally, the guidelines should make it clear that there is no need for separate recommendations for children with malaria (even those with high parasite burdens), SCD or poor nutritional status. As the trial demonstrated, a high proportion of children presenting with severe anaemia in many of our present study sites had undiagnosed SCD. The group recommended, therefore, that admission to hospital with severe anaemia should prompt clinicians to test for SCD so that long-term follow-up and infection prophylaxis can be put in place.

## Supplementary Material

Supplemental File

## Figures and Tables

**Table I:** Blood dosing chart for whole blood components.*

Weight, kg		20 ml/kg	Component† Volume	Min, ml/kg	Mid, ml/kg	Max, ml/kg	30 ml/kg	Component† Volume	Min, ml/kg	Mid, ml/kg	Max, ml/kg
5	6	100	100	16·7	18·2	20·0	150	150	25·0	27·3	30·0
6	7	150	150	21·4	23·1	25·0	200	2 x 100	28·6	30·8	33·3
7	8	150	150	18·8	20·0	21·4	200	2 x 100	25·0	26·7	28·6
8	9	150	150	16·7	17·6	18·8	250	250	27·8	29·4	31·3
9	10	200	2 x 100	20·0	21·1	22·2	300	2 x 150	30·0	31·6	33·3
10	11	200	2 x 100	18·2	19·0	20·0	300	2 x 150	27·3	28·6	30·0
11	12	200	2 x 100	16·7	17·4	18·2	350	250 + 100	29·2	30·4	31·8
12	13	250	250	19·2	20·0	20·8	400	150 + 250	30·8	32·0	33·3
13	14	250	250	17·9	18·5	19·2	400	150 + 250	28·6	29·6	30·8
14	15	300	2 x 150	20·0	20·7	21·4	450	Adult pack	30·0	31·0	32·1
15	16	300	2 x 150	18·8	19·4	20·0	450	Adult pack	28·1	29·0	30·0
16	17	300	2 x 150	17·6	18·2	18·8	500	Adult pack	29·4	30·3	31·3
17	18	350	250 + 100	19·4	20·0	20·6	500	Adult pack	27·8	28·6	29·4
18	19	350	250 + 100	18·4	18·9	19·4	550	Adult + 100	28·9	29·7	30·6
19	20	400	150 + 250	20·0	20·5	21·1	600	Adult + 150	30·0	30·8	31·6
20	21	400	150 + 250	19·0	19·5	20·0	600	Adult + 150	28·6	29·3	30·0
21	22	400	150 + 250	18·2	18·6	19·0	650	Adult + 150	29·5	30·2	31·0
22	23	450	Adult pack	19·6	20·0	20·5	650	Adult + 150	28·3	28·9	29·5
23	25	450	Adult pack	18·8	19·1	19·6	700	Adult + 250	29·2	29·8	30·4
>25		450	Adult pack	18·0	18·4	18·8	700	Adult + 250	28·0	28·6	29·2

Shaded rows are WHO standard weight bands.

* If whole blood packs are not available use red cell concentrate (RCC)/packed cells at half the volume, e.g. 20 ml/kg whole blood is equivalent to 10 ml/kg RCC.

† If multiple blood components are needed then pragmatically these can be obtained from multiple donors if a single donor component is insufficient.

**Table II:** What were the indications for second transfusions by volume randomisation and by fever status?

	Second transfusions for those randomised to transfusion arms, *n* (%)				
	Overall	20 ml with	20 ml without	30 ml with	30 ml without
Indication (at any time during admission)	(*n* = 496†)	fever* (*n* = 123)	fever (*n* = 177)	fever* (*n* = 83)	fever (*n* = 113)
New Hb <40 g/l	185 (37)	45 (37)	82 (46)	18 (22)	40 (35)
New severity signs‡ and Hb 40-60 g/l	9 (2)	3 (2)	1 (<1)	2 (2)	3 (3)
Known SCD or haemoglobinuria and Hb 40-60 g/l	206 (42)	56 (46)	63 (36)	37 (45)	50 (44)
Not per protocol§	96 (19)	19 (15)	31 (18)	26 (31)	20 (18)

Hb, haemoglobin; SCD, sickle cell disease.

* Axillary temperature ≥37·5°C.

† 497 transfusions were reported in the original trial paper but following publication it was found that one of those transfusions was of a negligible amount.^14^

‡ Severity sign (impaired consciousness and or increased work of breathing).

§ No recorded severity signs (impaired consciousness or increased work of breathing), no SCD or haemoglobinuria and Hb ≥40 and <60 g/l.
