# Supplementary material for: Transfusion management of severe anaemia in African children: a consensus algorithm
Source: Br J Haematol. Author manuscript; Available in PMC 2021 Jul 19. (PMC7611319; doi:10.1111/bjh.17429)

Algorithm Supplement

Figure S1: 30mls vs 20mls comparison by fever status at screening and sickle cell status

Subgroup	30mls/kg	20mls/kg	30mls/kg: 20mls/kg HR (95% CI)
SICKLE CELL FROM GENOTYPE*			
AA or AS - no fever	3/235 (1.3%)	10/225 (4.4%)	0.29 (0.08-1.04)
AA or AS - fever	20/419 (4.8%)	13/399 (3.3%)	1.47 (0.73-2.96)
SS - no fever	21/693 (3.0%)	44/720 (6.1%)	0.49 (0.29-0.83)
SS - fever	7/189 (3.7%)	1/191 (0.5%)	7.11 (0.88-57.8)
HAEMOGLOBINUREA AT ADMISSION			
Yes - no fever	4/180 (2.2%)	14/196 (7.1%)	0.31 (0.10-0.94)
Yes - fever	6/110 (5.5%)	2/104 (1.9%)	2.87 (0.58-14.20
No - no fever	20/783 (2.6%)	42/784 (5.4%)	0.48 (0.28-0.81)
Yes - fever	25/525 (4.8%)	14/514 (2.7%)	1.71 (0.89-3.29)
AGE OF CHILD AT SCREENING			
≤1 year old - no fever	7/286 (2.5%)	18/292 (6.2%)	0.39 (0.16-0.94)
≤1 year old - fever	15/234 (6.4%)	9/244 (3.7%)	1.75 (0.77-4.01)
2-3 years old - no fever	6/289 (2.1%)	18/265 (6.8%)	0.30 (0.12-0.76)
2-3 years old - fever	7/177 (4.0%)	2/172 (1.1%)	3.45 (0.72-16.6)
4+ years old - no fever	11/388 (2.8%)	20/423 (4.7%)	0.60 (0.29-1.25)
4+ years old - fever	9/224 (4.0%)	5/202 (2.5%)	1.62 (0.54-4.83)

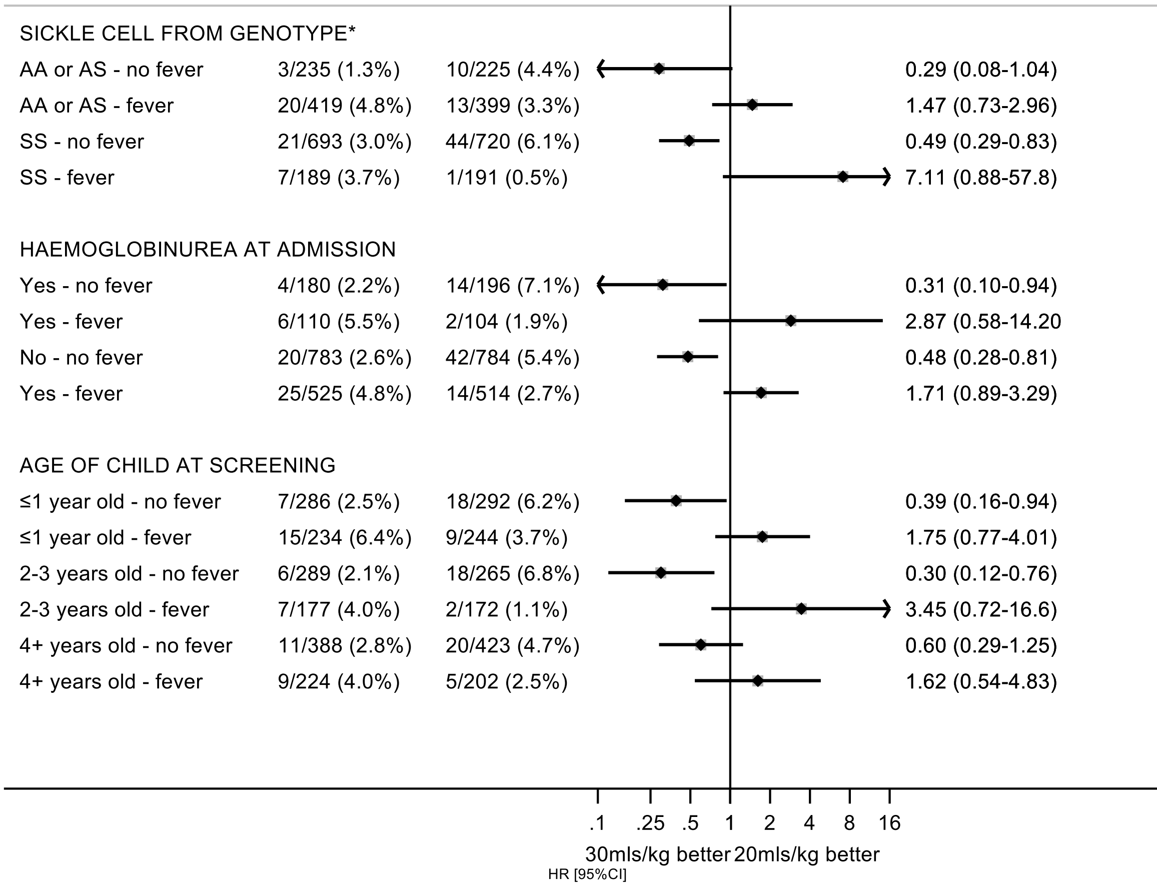

From genotype status: Hb AA (normal Hb) Hb AS (Sickle Cell Trait) Hb SS (Sickle Cell Disease)
NB: p=0.34 for interaction with fever and sickle status. p=0.83 for interaction with fever and haemoglobinuria at admission. p=0.63 for interaction with fever and age

Figure S2 Stability of Temperature following screening/randomisation

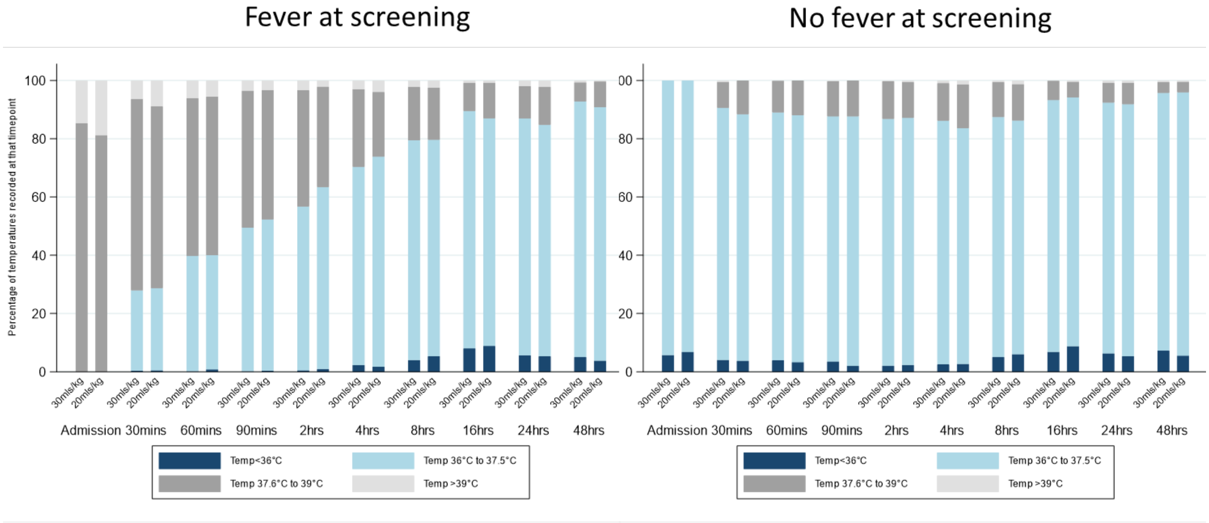

Table S1 Suspected overload Events: endpoint review

	Event name	Grade	Transfusion amount	Relatedness to transfusion	Relatedness to transfusion volume	Clinical review form
1	Transfusion associated circulatory overload	3	30mls/kg			Not considered randomisation related*
2	Anaemia with clinical symptoms+pneumonia	5	20mls/kg	Unlikely	Unlikely	Not considered PO/TRALI/TACO
3	Death	5	20mls/kg	Unrelated	Unrelated	Not considered PO/TRALI/TACO
4	Anaemia with no clinical symptoms	3	30mls/kg	Unrelated	Unrelated	Not considered PO/TRALI/TACO
5	Pulmonary oedema	3	20mls/kg	Unlikely	Unlikely	Pulmonary oedema

* the childs' management by attending clinical team (post 48 hours) resulted in the child received large volume of transfused blood to target a Hb level of 10 g/dl as the child was a newly diagnosed SCD and the clinical team suspected lung sequestration. The child developed signs of fluid overload.

Figure S3 Heart rate and respiratory rate over time from beginning of first transfusion.

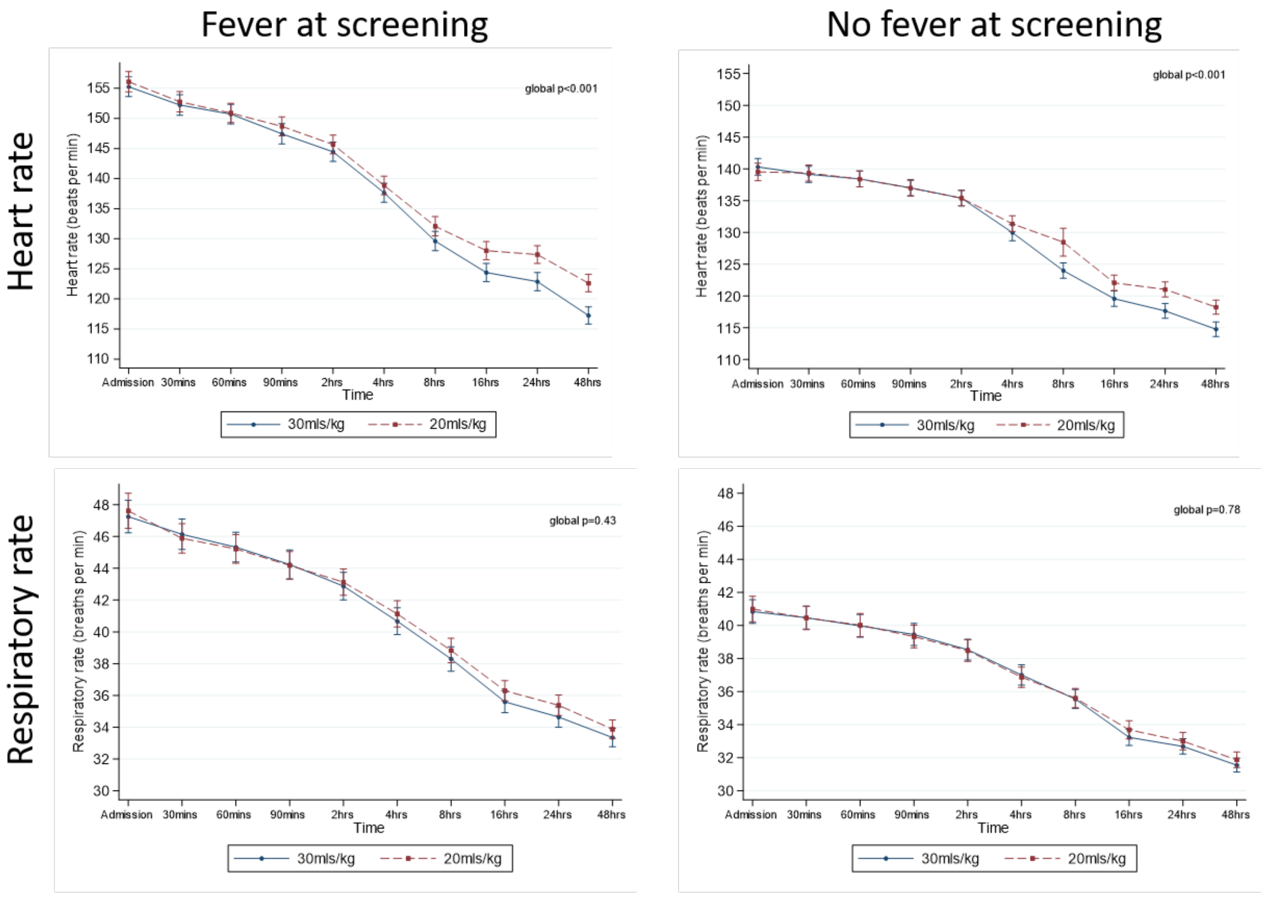

Supplement: Supplemental File [file EMS130214-supplement-Supplemental_File.pdf]
